# Pododynia: Its Causes and Significance

**Published:** 1881-04

**Authors:** T. B. Curtis

**Affiliations:** Boston Society for Medical Improvement


### PODODYNIA: ITS CAUSES AND SIGNIFICANCE.

By T. B. CURTIS, M.D.

Read before the Boston Society for Medical Improvement, March 28, 1881.

The word *pododynia*, or *podalgia*, is here used, for the sake of brevity, to designate certain painful affections of the feet, in which pain, spontaneous or provoked by contact and pressure, exists independently of any physical signs, visible or tangible, of local organic disease of the part, whether inflammatory or traumatic. A puzzling and refractory case, encountered in private practice a few years ago, furnished the occasion and incentive for studying the different varieties of painful affections of the feet, in which mere *pain*, apparently independent of any gross lesion, constituted the main, or only, symptom complained of by the patient. I was thus led to rehearse and investigate the numerous etiological conditions in which pododynia, as above defined, is liable to occur; and the following short account of what was to be found in the medical literature touching this comparatively unimportant topic, represents merely a contribution to the elucidation of the semiology of pain in the foot.

The symptom in question, especially when limited to the purely subjective phenomenon pain, and unaccompanied by any discoverable local lesion, may appear a trivial matter; nevertheless, a chronic, long lasting pain or tenderness of the foot is often so disabling, by interfering with locomotion, that the importance of correct diagnosis, prognosis, and treatment is by no means commensurate with the apparent insignificance of the ailment complained of. Moreover, although the causes of pain in foot are very diverse, and their connections with this local manifestation is often remote and obscure, pododynia, considered only as a symptom, has not unfrequently a real diagnostic interest and value; and is, therefore, I think, worthy of attention, from a semeiological point of view. Without further apology, then, for dwelling upon a small topic, I will first give a short account of my case, and then add such information as I have been able to collect on the subject of the disease from which my patient was suffering, as well as concerning the other forms of pain in the foot occasionally encountered in the practice:

*Case.* A large, heavy man, about forty-five years of age, stout, and somewhat plethoric in appearance, but in other respects looking robust and healthy, came to my office in August, 1877, complaining of a pain in the right heel. In answer to questions, he informed me that for some years before he had occasionally noticed urinary deposits, "brick-dust," together with slight attacks of temporary irritation of the bladder. He had also had "rheumatic" pains of uncertain character. No other morbid antecedents could be elicited. The pain was first felt about six weeks before his visit. He remembered having slipped and struck his heel, a few days previously, but this accident seemed a trivial one, and he attached, at the time, little or no importance to it.

At the time of his visit, strong pressure showed the existence of a tender spot on the sole of the right heel, about two inches from the posterior extremity of the *os calcis*. No other symptom was detected by a careful examination. The appearance of the part was perfectly normal, heat, redness and swelling being entirely absent. The patient was quite free from pain, so long as he kept quiet, and he walked into my office without limping; but when he went even a moderate distance on foot, the pain soon became sufficiently acute to render further walking very uncomfortable, and caused a degree of lameness which hindered him very materially in the accomplishment of his bread-winning duties, the latter being those of a business-agent and errand runner, and keeping him a great deal on his feet. The diagnosis entered in my record of the case was "contusion; pain kept up by walking." I advised complete rest for a week, and the use of an alkaline mixture, in which twenty-five grains of citrate of potassium were to be taken thrice daily.

The patient returned two months later in the same condition as when first seen. The pain had ceased during abstinence from walking, recurring, however, as soon as he resumed his habitual occupations. Moreover, the other foot had been similarily affected, with a pain in the *tendo Achillis*. I then advised counter irritation by means of iodine, together with the internal use of iodide of potassium, in doses of five grains, thrice daily. I failed to see the patient again, but have since been told by a mutual acquaintance that he had remained without improvement. I also learned that before applying to me he had been under the care of one of our most experienced surgeons.

*Remarks.* Not long after the disappearance of this dissatisfied and unsatisfactory patient, I came across a description of his trouble in a book recently published by Dr. Gillette, a surgeon of the Paris Hospitals. In this work, Dr. Despres, of Paris, also a hospital-surgeon, is quoted as having bestowed the name of "policeman's disease" (*maladie des gardiens de la paix*, the latter designation being a euphemism which, after the downfall of the Empire, replaced the abhored term *sergent-de-ville*) upon a painful affection of the foot, originating in a deep-seated contusion of the adipose cushion which covers the *os calcis*, and perpetuated by pressure undergone during locomotion. This ailment has, it seemed, been first described, many years ago, in 1883, by Velpeau. It had also been more recently the subject of an essay by Dr. Fabre. According to Despres, whose cases were chiefly observed among the Paris policemen, the pain generally affects first the right heel. It diminishes or subsides altogether during rest, but recurs, with or without implication of the other heel, when walking is resumed or persisted in.

Such is the "policeman's disease" of the French. I once inquired of Dr. Samuel A. Green, who in his capacity of city physician, is often called upon to attend our sick or disabled policemen, whether among the latter he had ever met with such cases of painful heels. He had, however, had no such experience.

Pain in the heel, or sole, unattended by any objective symptoms, occurs under the following varied conditions:

(1.) As an irradiated, reflected, or so-called "sympathetic" pain, in cases of *urethral stricture*, it has been observed by Luxmoor, Brodie, and many others.

(2.) In cases of *vesical calculus*, "one of the most interesting examples of this," says Von Pitha, "occurred in the person of a colleague, Dr. Reisich, of Prague, who in 1857, was freed from a large stone by lithotripsy. . . . He indicated, as one of the most precise symptoms of the calculus, a sensation as if he were standing with his left foot on a red-hot plate. With each successive diminution of the size of the stone, by means of the operation, the circumference of this burning plate seemed to diminish, and at last only a small edge of the sole remained the seat of this reflected pain. This remnant, however, continued obstinate after I had, as I believed, removed all the detritus, and when exploration failed to detect anything within the bladder. In spite of my denial, the patient insisted determinedly that, according to his sensations, some small fragments still existed at the left side of the fundus of the bladder. I examined him again, and at the end of a few weeks found, at the spot which he had indicated, a small fragment concealed in a fold of the mucous membrane. When this was removed all pain in the sole disappeared, and the patient, for the first time, expressed himself as relieved."

(3.) Similar irradiated or reflected pains in the foot are occasionally experienced in *cysto-prostatitis* or inflammation of the neck of the bladder. A patient of mine, somewhat advanced in years, had suffered for several years from obstructive hypertrophy of the prostate, with complete retention of the urine, requiring the habitual daily use of the catheter, and complicated by chronic catarrh of the bladder. He had also had a phosphatic vesical calculus, which was removed by lithotrity, partly by Prof. Guyon in Paris, and partly by myself after the return of the patient to this country. He had for several months been afflicted with a most painful and harrassing complication, namely, a chronic cysto-prostatitis, liable to frequent acute or subacute exascerbations. A considerable degree of relief, by the way, of the distressing and almost ceaseless vesical tenesmus caused by this inflammatory affection accrued from the use of intra-prostatic injections of strong solutions of nitrate of silver—varying in strength from one in fifty to one in twenty parts of water—practiced with Guyon's injector, at intervals varying from three or four to six or seven days. In this patient, curiously enough, every paroxysm of prostatic pain, whether caused by movements in bed, by locomotion, by calls to urinate, by catheterism or by the applications of nitrate of silver, was and still is liable to be accompanied by a sharp, distinctly localized pain in the ball of the left foot, the sensation then felt being compared by him to that which would be caused by treading with the bare foot upon tacks or broken glass. The pain at the neck of the bladder was of the same description, as if due to the presence of a sharp, jagged foreign body, so as to lead to repeated explorations for calculus under ether; this pain, also, is left sided, being referred to the left side of the vesical neck and rectum.

(4.) In *cystalgia* or neuralgia of the neck of the bladder, Von Pitha himself, suffering from this neurosis, was actually sounded for stone as often as five times, by his own desire, so closely were the symptoms of vesical calculus simulated by his cystalgic paroxysms. A very severe neuralgia at the same time also affected the heel, the pain, at its moments of greatest severity, taking on exactly the sensation that would have been experienced if the periosteum were being forcibly separated from the *os calcis*. "The pains are so intense," says Von Pitha, "and the deception so complete, that I am under the conviction that if the subperiosteal excision of the *os calcis* were being executed, the pains would only resemble in nature and severity those which I suffer during the acute paroxysm."

(5.) In gout, besides the pain attending the acute inflammatory attacks of dermatitis, lymphangitis and cellulitis, caused by the deposits of biurate of sodium, sharp pains in the feet, particularly in the heel, at times occur without any objective symptoms. Sir James Paget cites pain in the heel as one of the many lesser signs of gout. "Of course," he says, "it was not meant that no further inquiries should be made, but only that pain at the heel ought to suggest gout, and lead to further questions on the point. Similarily, pain in the tendo Achillis, especially in elderly persons, who had sustained no injury, was due, with very few exceptions, to some degree of gout." Elsewhere he says, "I cannot remember to have heard any patient complaining of spontaneous pain in his tendo Achillis, except such as I knew to be by inheritance disposed to gout or a lithic acid diathesis. Pain in the heel of an elderly person has, generally, the same meaning. . . . . 'Burning soles' and the less frequent 'burning palms' generally signify a gouty constitution or one closely allied to it, and so do the sensations of hot, tingling and burning patches of the skin of the thighs, without external appearance of redness or eruption." Dr. Dyce Duckworth, of Saint Bartholomew's Hospital, to whose extensive and pains-taking observations we owe much valuable information touching the protean signs of the gouty constitution, testifies to the same effect as follows: "Deep-seated pain in the heel has been recognized as of gouty origin. The sensation is compared to the feeling of a foreign body being implanted there, such as a bullet. And it is noteworthy that this is sometimes a symptom of a renal calculus which may itself be the outcome of a gouty taint. The pain is sometimes distinctly in the tendo Achillis." Charcot mentions the tendo Achillis as one of the ligamentous structures in which uric acid is liable to be deposited in overly gouty subjects. A discussion took place a few years ago in one of the medical societies of Paris, on pain in the heel, or talalgia, and its treatment, in which several speakers took part. It was agreed that the pain was often due to a gouty or rheumatic diathesis, and the use of salicyate of soda, in daily doses of one or two drachms, was said by Bucquoy, Panas and Despres to be often efficacious.

The burning soles and palms, alluded to by Sir James Paget, may, perhaps, be considered more or less analogous to the distressing and at times painful affection of the extremities, recently described by Dr. S. W. Mitchell, under the title of a Rare Vaso-Motor Neurosis. In this chronic and most refractory disease, however, for which the descriptive name of *erythromelagia* is proposed by Dr. Mitchell, the subjective sensation of heat or scalding is accompanied by a marked temporary flushing or hyperæmia of the affected parts, with redness and warmth of the surface. This affection, therefore, cannot properly be included in the category of purely subjective ailments considered in this paper.

A patient of my own, an elderly lady, coming from a distinctly gouty stock, who had suffered severely somewhat over a year ago from gall stones, with severe hepatic colic and jaundice, and who has lately placed herself, by my advice, under the care of Dr. J. C. White, on account of a persistent and most distressing generalized pruritus, has also suffered, since last October, what I take to be erythromelalgia, namely, paroxysms of painful burning of the feet accompanied, according to her report, by flushing of the skin, and occurring mostly at night in bed.

(6.) *Renal calculus* occasionally gives rise to a pain irradiated to the heel, as stated by Dr. Dyce Duckworth in the passage quoted above.

(7.) In *gonorrhœa* pain in the heel is described by Fournier, Panas, and others, as an occasional manifestation, more or less akin to the various forms and localizations of so called "gonorrhœal rheumatism," which affects the articular and tendinous synovial membranes and the bursæ. It was first described as affecting the heel in this disease by Swediaur.

(8.) In *syphilis*, also, according to Fournier, the same phenomenon is met with. Here, as in gonorrhœa, the pain is attributed by him to a bursitis.

(9.) In *locomotor ataxia* the heel may be the first or, for a while, the principal seat of the lancinating or boring pains, characteristic of the first stages of that strange disease. A patient whom I had occasion to see a few years ago, and whom I sounded for stone, at the request of his medical attendant, was suffering from occasional painful paroxysms of vesical and rectal tenesmus For several years previously he had experienced sharp boring pains (the *douleurs terebrantes* of Charcot) which had first attacked the heel. He subsequently developed ataxic symptoms, with staggering gait. Dr. Seguin, describing the symptoms of locomotor ataxia, says that "patients usually give up in despair the attempts to show you every spot in which they have suffered, and they generally indicate as foci of pain the heel, instep and thigh." Dr. Buzzard also expressly mentions the heel as frequently the seat of pain in tabes.

(10.) Lastly, an obscure form of pain in the feet has been very briefly described by Prof. S. D. Gross, under the name of *pododynia.* It shows itself in the form of great tenderness to pressure, and is met with in certain sedentary classes of artisans, particularly in tailors.

Such, mainly, are the manifold conditions under which the purely subjective symptom, *pain in the foot*, may occur. This little ailment is not unfrequently as puzzling and refractory to the medical attendant as it is annoying and disabling to the patient. This consideration, will I trust, be accepted in justification of the attention which I have ventured to bestow upon the subject.

